# A core outcome set for localised prostate cancer effectiveness trials: protocol for a systematic review of the literature and stakeholder involvement through interviews and a Delphi survey

**DOI:** 10.1186/s13063-015-0598-0

**Published:** 2015-03-04

**Authors:** Steven MacLennan, Hendrika J Bekema, Paula R Williamson, Marion K Campbell, Fiona Stewart, Sara J MacLennan, James MO N’Dow, Thomas BL Lam

**Affiliations:** Academic Urology Unit, University of Aberdeen, Cornhill Road, Aberdeen, UK AB25 2ZD; Department of Critical Care, University of Groningen, University Medical Center Groningen, Hanzleplein, Groningen The Netherlands 9700 RB; Department of Biostatistics, University of Liverpool, Crown Street, Liverpool, UK L69 3BX; Health Services Research Unit, University of Aberdeen, Cornhill Road, Aberdeen, UK AB25 2ZD; Department of Urology, Aberdeen Royal Infirmary, Foresterhill Road, Aberdeen, UK AB25 2ZD

**Keywords:** Core outcome set, Systematic review, Semi-structured interviews, Delphi, Consensus methods, Treatment for localised prostate cancer

## Abstract

**Background:**

Prostate cancer is a growing health problem worldwide. The management of localised prostate cancer is controversial. It is unclear which of several surgical, radiotherapeutic, ablative, and surveillance treatments is the most effective. All have cost, process and recovery, and morbidity implications which add to treatment decision-making complexity for patients and healthcare professionals. Evidence from randomised controlled trials (RCTs) is not optimal because of uncertainty as to what constitutes important outcomes. Another issue hampering evidence synthesis is heterogeneity of outcome definition, measurement, and reporting. This project aims to determine which outcomes are the most important to patients and healthcare professionals, and use these findings to recommend a standardised core outcome set for comparative effectiveness trials of treatments for localised prostate cancer, to optimise decision-making.

**Methods/Design:**

The range of potentially important outcomes and measures will be identified through systematic reviews of the literature and semi-structured interviews with patients. A consultation exercise involving representatives from two key stakeholder groups (patients and healthcare professionals) will ratify the list of outcomes to be entered into a three round Delphi study. The Delphi process will refine and prioritise the list of identified outcomes. A methodological substudy (nested RCT design) will also be undertaken. Participants will be randomised after round one of the Delphi study to one of three feedback groups, based on different feedback strategies, in order to explore the potential impact of feedback strategies on participant responses. This may assist the design of a future core outcome set and Delphi studies. Following the Delphi study, a final consensus meeting attended by representatives from both stakeholder groups will determine the final recommended core outcome set.

**Discussion:**

This study will inform clinical practice and future trials of interventions of localised prostate cancer by standardising a core outcome set which should be considered in comparative effectiveness studies for localised prostate cancer.

**Electronic supplementary material:**

The online version of this article (doi:10.1186/s13063-015-0598-0) contains supplementary material, which is available to authorized users.

## Background

Prostate cancer is the second most common male cancer worldwide (an estimated 1.1 million cases diagnosed in 2012), and the fourth most common cancer overall [1]. It is the fifth most common cause of cancer death in males worldwide (307,000 deaths in 2012), and about 70% of registered cases (759,000) occur in more developed regions (Western and Northern Europe, Australia, New Zealand, North America, Polynesia, and the Caribbean) [1]. Incidence and mortality rates are generally high in predominantly black populations and very low in Asia, suggesting a possible genetic determinant [2-4]. The worldwide burden is expected to grow due to global population growth and ageing [3]. In contrast to the rising incidence, mortality rates have decreased in most high resource settings (including North America, Northern and Western Europe, and Oceania).

Prostate cancer is the most common cancer in men in the United Kingdom [5]. In 2010, nearly 41,000 new cases were reported; a quarter of all new male cancer diagnoses [5]. The incidence of prostate cancer is rising [5,6] due to increasing use of prostate specific antigen (PSA) testing [5-7] and the ageing population [8]. The largest rise in incidence is in men with localised prostate cancer [5]. Prostate cancer causes 13% (10,721) of cancer-related deaths each year [9]. The total costs for prostate cancer for the United Kingdom in 2009 (encompassing treatment costs for surgery, radiotherapy, hospital and community care, premature deaths, time off work, and unpaid care to patients by family and friends), were estimated at approximately £800 million per annum [10]. Consequently, prostate cancer represents a significant healthcare burden for the United Kingdom.

In the United Kingdom, more than 4,000 radical prostatectomies are carried out annually [11]. Of these, the majority are open procedures, although laparoscopic and robotic-assisted techniques are increasingly being performed [12-15], which has added to the ongoing dilemma for surgeons, patients, and health services when making treatment choices. Radical prostatectomy is associated with significant morbidity and incurs substantial health service costs. Consequently, there is an urgent need for high quality evidence, including well designed and properly conducted randomised controlled trials (RCTs) comparing all the major interventions for localised prostate cancer. Some of these data will be forthcoming, as studies comparing surgery versus non-surgical interventions, such as Prostate Testing for Cancer and Treatment (ProtecT) [16] and Prostate Cancer Intervention Versus Observation Trial (PIVOT**)** [17], are increasingly being undertaken. The 10-year median follow-up period from the PIVOT study [17] suggests that radical prostatectomy does not significantly reduce all cause or prostate cancer specific mortality compared with observation, especially in low risk patients, although there is a suggestion from the subgroup analysis that surgery may be of benefit for intermediate and high risk patients.

Currently completed and ongoing trials are not as helpful as they could be, due to lack of standardisation of outcome definition, collection, and reporting [18,19]. These outcome reporting and definition problems have been highlighted as a hindrance in evidence synthesis in a number of localised prostate cancer intervention effectiveness systematic reviews [18,20-24]. Whilst trials of prostate cancer interventions routinely report process-related outcomes (such as duration of hospital stay) and clinical outcomes (such as survival, incontinence, or erectile dysfunction), the heterogeneity of reported outcomes hinders comparisons of alternative interventions for decision-making by stakeholders [20]. Oncological outcomes such as survival, cancer progression, and cancer recurrence are broadly similar across the different treatment options in the short term [21]. Hence, the choice between treatments for patients is likely to also be driven by other considerations such as adverse events, impact on quality of life, patient experience of care, patient satisfaction with care, and speed of return to productivity or routine activities [25,26]. For clinicians, the choice may be influenced by learning curve issues and outcomes such as positive margin [27] and PSA recurrence rates [28]. The lack of consistency and clarity of what outcomes should be measured and reported hampers decision-making by all stakeholders.

For researchers, the design of RCTs is compromised at various stages, including sample size calculations, and potentially important outcomes not being measured, analysed, or reported, resulting in outcome reporting bias [19]. For the NHS in the United Kingdom, the lack of reliable cost-effectiveness data hinders decision-making. For patients and healthcare professionals, shared decision-making in terms of understanding the risks and benefits of the different treatment options is compromised, informed consent is hampered, clinical governance becomes problematic, and participation in RCTs becomes difficult. Hence, there is an urgent need to identify a core outcome set of universal importance, which reflects the perspectives of all stakeholders. A core outcome set is defined to be ‘an agreed standardised set of outcomes that should be measured and reported, as a minimum, in all clinical trials in specific areas of health or health care’ [29].

The Core Outcome Measures in Effectiveness Trials (COMET) initiative was launched in January 2010 [30] to address the lack of standardised core outcome measures in clinical trials. COMET has searched the literature to identify studies which have developed outcome sets in a variety of conditions, and maintains a database [31,32]. The database includes 12 studies relevant to prostate cancer, with six studies focusing exclusively on localised prostate cancer. Although all of these studies make recommendations for a localised prostate cancer-specific core outcome set, all have various methodological flaws, such as: not including patients in the consultation process [33,34], relying solely on literature reviews for the generation and recommendation of outcomes with no primary research to propose or prioritise outcomes [34-37], focusing on treatment decisions by patients and ignoring post-intervention experience and outcomes suggested by clinicians [36], or including only a clinical expert and/or guideline panel participants and no patients [34,35,37].

One study by the International Consortium for Health Outcomes Measurement (ICHOM) [38,39] did include two patient representatives, but the methods for prioritising and achieving consensus on which outcomes to include and how patients were involved were not transparent. Furthermore, ICHOM’s focus is toward routine data collection in clinical practice, with a view to comparing healthcare providers’ outcomes in a competitive sense, as opposed to providing a core outcome set for use in effectiveness trials. The present research represents a robust attempt at involving healthcare professional and patient stakeholders, and developing a localised prostate cancer-specific core outcome set for use in effectiveness trials using standardised and validated methodology, in close collaboration with COMET. Furthermore, we have included a nested RCT within the Delphi phase of this research project in order to investigate how differences in how outcomes are scored might be affected by the feedback participants have access to. This may assist COMET and other core outcome set development projects in designing future Delphi projects.

### Aims and objectives

#### Aim

The overall aim of this project is to develop a core outcome set for localised prostate cancer effectiveness trials which recommends what outcomes should be measured, and reflects the interests of patients and healthcare professionals, in order to facilitate decision-making.

#### Objectives

The specific objectives are: (1) to identify a list of outcomes from published studies reporting on any therapeutic intervention for localised prostate cancer; (2) to determine the heterogeneity of outcome definitions, and the number of different measuring instruments used and the specific ways in which they differ; (3) to identify a list of potentially important outcomes reported by men who have been treated for localised prostate cancer in order to augment the list generated from (1); (4) to prioritise and reach consensus regarding the most important outcomes from the perspective of patients and healthcare professionals into a core outcome set; and (5) to investigate how differences in Delphi study outcome scoring might be affected by the feedback participants have access to in order to assist the design of future Delphi studies.

## Methods/Design

The project will be divided into two distinct phases: (1) generation of a list containing all possible relevant outcomes (systematic review of the literature and semi-structured interviews with patients); and (2) prioritisation of important outcomes from stakeholder groups (patients and healthcare professionals; Delphi study for each group), followed by integration of outcomes into a core outcome set (consensus group meeting).

### Phase one: generation of a list containing all possible relevant outcomes

#### Systematic review

Research question: What are the outcomes reported in studies assessing the effectiveness of interventions for localised prostate cancer?

##### Study overview and method

This study will consist of a systematic review of studies of the effects of surgery (open, laparoscopic, and robotic-assisted radical prostatectomy), radiotherapy, brachytherapy, and active monitoring for localised prostate cancer. Patient care pathways (see Additional file 1) have been established with consensus from international clinical content experts; these will provide a conceptual framework to identify potential outcomes [40].

##### Types of studies

We intend to limit included studies to those that are likely to influence clinical practice. For this reason, the Oxford Centre for Evidence Based Medicine’s guidelines regarding hierarchy of evidence [41] will be adhered to. Accordingly, where the list of identified studies includes more than one RCT for an intervention and comparator pair, we will include only the RCT. When there is only one or there are no RCTs in the identified studies list, we will include non-randomised comparative studies, and where there are only single-arm case series (which are more likely for newer interventions or technologies) we will include these. Where non-randomised studies have been included and RCTs are found in subsequent updates, we will retain the non-randomised studies. Ongoing trials (identified in trial registers) will also be included. It is possible that including all types of study design would yield a larger number of outcomes that could perhaps reflect the views of all stakeholders more comprehensively. However, subsequent parts of the research, such as patient interviews (outlined below), and the Delphi study (outlined below), provide ample opportunity to ensure all stakeholders’ views regarding potentially important outcomes are considered.

##### Types of interventions

Interventions considered for this review are: surgery (open, laparoscopic, or robotic, incorporating any approach and technique); external beam radiotherapy (EBRT), three-dimensional conformal radiotherapy (3D-CRT), or intensity modulated radiotherapy (IMRT), incorporating any dose or schedule; brachytherapy (permanent or temporary seed implantation, incorporating any dose or schedule); cryotherapy; high intensity focussed ultrasound (HIFU) active surveillance or monitoring; and watchful waiting or observation.

##### Types of participants

The participants are men of any age with clinically localised prostate cancer (defined as cT1-2c N0 M0, according to the TNM classification of malignant tumours) [42].

##### Exclusion criteria

In studies where more than 20% of the population are not clinically localised (>cT2c, or N+ or M+), the study will be excluded. Studies of dietary interventions will be excluded. Studies with fewer than 10 patients per intervention arm will be excluded because they are unlikely to influence practice.

##### Search methods for identification of studies

The review will be reported in accordance with the Preferred Reporting Items for Systematic Reviews and Meta-Analyses (PRISMA**)** guidelines [43]. We will include studies identified from four existing systematic reviews which have similar inclusion criteria to this research [20,28,44]. The excluded studies lists from these reviews will be screened to minimise the possibility that some of these studies were excluded on the basis of not reporting outcomes of interest. We will not re-screen original abstract lists because it is very unlikely that reviewers would have excluded on the basis of outcome during abstract screening. Update searches of the existing reviews will also be performed and any further studies which meet inclusion criteria (outlined below) will be included.

##### Eligibility of studies

Two review authors (SM and TBLL) will independently assess the abstracts returned from the searches. Full texts of all potentially relevant studies will be obtained. Any studies not meeting inclusion criteria will be excluded. Where a resolution cannot be reached, a third review author (JMOND) will be consulted.

##### Data extraction

Data will be extracted independently by two review authors (SM and HB) and checked by a third author (TBLL). SM and TBLL will then review the extracted data to assess consensus and ensure all outcomes have been identified. The following data will be extracted from each study: study type, author details, year and journal of publication, intervention(s) under investigation, each effectiveness outcome reported, whether the outcome was defined or not, the definition used, the indicators and/or tool(s) used to operationalize or measure the outcome, the time point or period of outcome measurement, and how the outcome was reported. Disagreement will be resolved through discussion; where a resolution is not possible a third reviewer (JMOND) will be consulted. We will contact study authors to identify any unclear and/or unavailable data.

##### Data analysis and presentation

The data will be entered into Microsoft Excel in order to aid tabulation and analysis. Outcomes will be grouped under domains following a review of the outcomes by TBLL and SM. Outcomes regarded as ‘harmful effects’ will be grouped within the domain ‘Adverse Events’. The outcome domains and included outcomes will be reviewed by the Study Advisory Group to assess the suitability of the domain name and outcomes grouping. We will then evaluate how many outcomes have been used to reflect each domain, and how many different definitions and measurements were used.

### Semi-structured interviews with individual patients

Research question: What are the outcomes patients regard as potentially important following treatment for localised prostate cancer?

#### Study overview and method

Semi-structured interviews with patients who have had treatment for localised prostate cancer. Semi structured interviews have been used effectively to ascertain the patient perspective in previous COMET and Outcome Measures in Rheumatology (OMERACT) studies [45,46]. We anticipate that intervention type, age at intervention, and time since intervention will influence the outcomes patients regard as potentially important. We will purposively sample to cover a wide range of available interventions for localised prostate cancer, age at intervention, and time since intervention. An initial analysis sample of 15 will be recruited because there are relatively few stratification factors (akin to independent variables), and we will use a stopping criterion of three [47]. That is, a wide range of idiosyncratic and common outcomes (reported by at least two patients) are anticipated to be discernible within 12 interviews; if no new common outcomes emerge after interview 15, data saturation can be demonstrated. If new common outcomes are identified within the three additional interviews, we will conduct a further three interviews, and so on until no new outcomes are reported.

Participants will be sampled to ensure diversity of treatment type, time since intervention, and age at time of intervention. Each participant will be given an information booklet outlining the study. Following this, consent will be obtained from each participant by attaining their signature on a consent form which outlines that they understand the purpose of the study, the uses their data will be put to, and their right to withdraw at any point. Interviews will be audio-recorded and transcribed in full. Coding of the data will proceed using a thematic analysis approach and emerging themes identified, whilst paying attention to the number of idiosyncratic and shared outcomes reported. The data will be checked for validity and contextual accuracy to compile a list of important outcomes.

##### Analysis of semi-structured interviews

Audio recordings of the semi-structured interviews will be fully transcribed, stored, and analysed using NVivo10 software (QSR International, Burlington, Massachusetts, United States) and themes will be derived from issues raised by participants. To aid analysis, an adapted framework method of data management [48] will be used in order to chart the coded data. This will also enable the identification of additional new codes. In this way, important information will be identified from a large amount of data in a structured fashion, which will enable recognition of when data saturation has been reached and will inform stopping criterion strategy.

### Consultation exercise

Research question: From the list of outcomes identified from the systematic review and semi-structured interviews with patients, what are the outcomes that should be entered into phase two for further study?

#### Study overview and method

Consultation exercise with patients. The list of outcomes identified from the literature review and from the semi-structured interviews with patients will be combined, and the objective of the consultation exercise is to ratify the outcomes to ensure clear and efficient meanings are given, and that there is no duplication. This ratified comprehensive list of outcomes will be entered into phase two. The consultation exercise will include the research team (TBBL, JMOND, and SM); the steering group (Professor Marion Campbell, Professor Craig Ramsay, Professor Luke Vale, Professor Paula Williamson, and Professor Vikki Entwistle), and a focus group with seven patients (purposively sampled for a variety of treatment types). Each participant will be given an information booklet outlining the study. Following this, consent will be obtained from each participant by attaining their signature on a consent form which outlines that they understand the purpose of the study, the uses their data will be put to, and their right to withdraw at any point. A separate healthcare professional group will not be consulted separately because their views have been covered in the systematic review. Furthermore, one of the project researchers (TBLL) and one of the study advisory group members (JMOND) are consultant urologists and can comment on the list from a clinical perspective.

#### Phase two: prioritisation of important outcomes from each stakeholder group and integration of outcomes into a core outcome set

Research question: What are the most important outcomes for each key stakeholder group?

#### Study overview

A survey of key stakeholder opinions using Delphi methodology will be conducted. The list of potential outcomes finalised from phase one will be formatted into ‘items’, with a response designed to allow the participants to rate each of the items’ value for the final core outcome set, with high scores indicating the importance of inclusion. An online questionnaire will be developed for the Delphi process and piloted for each key stakeholder group (patients and healthcare professionals, consisting of clinicians and specialist nurses). It is anticipated that each Delphi process will consist of three rounds, with participants numbering up to 150 for the patient and healthcare professional groups. Although there is no consensus regarding the appropriate sample size used in Delphi methodology, we will draw upon the experience from previous Delphi studies [46,49,50], and also be guided by COMET. Items addressing similar constructs will be worded and phrased accordingly for both groups of participants to ensure understanding. Patients will be locally recruited from the north of Scotland prostate cancer patient support group (Urological Cancer Charity (CAN)) and nationally from patient-led support groups that are members of the umbrella organisation Prostate Cancer Support Federation (PCSF).

The healthcare professional group will comprise of localised prostate cancer specialist nurses, consultant clinical and medical oncologists, and consultant urological surgeons currently undertaking all types of radical prostatectomy or other therapies for localised prostate cancer (including cryotherapy and HIFU). They will be identified and recruited through the British Association of Urological Surgeons (BAUS), and the group will be complemented by British and international experts (from Europe and the United States) in open retropubic, laparoscopic, and robotic radical prostatectomy, and minimally invasive ablative therapies (including cryotherapy and HIFU), with whom our group has collaborated with on several National Institute for Health Research, Health Technology Assessment (NIHR HTA) Programme-commissioned systematic reviews on the clinical effectiveness of interventions for localised prostate cancer [20,28]. Each participant will be emailed information outlining the study. Informed consent will be assumed if participants register for the online Delphi questionnaire and submit their answers.

A nested RCT will also be included within the Delphi study. Randomisation into one of three groups will be performed when a participant logs on to round two. From their round one data it will be known which of the two stakeholder groups they are in (effectively stratifying by stakeholder group). It is intended that variable block randomisation will be used. Group one will have access to their own stakeholder group’s scores only as feedback, group two will have access to their own stakeholder group’s and the other stakeholder group’s scores, and group three will have access to both stakeholder groups’ scores combined. The main outcome of interest for the nested RCT is the difference between elements of the core outcome sets developed in each randomised group. To maximise the information gained from the multiple consensus exercises in the final core outcome set, all results will be considered at the final consensus meeting. If the trial finds no obvious differences between the approaches, it would not be inferred that they are intrinsically the same, or that this finding could be applied in other settings (since it could be due to inadequate power or homogeneity of opinion in the studied area); but if important differences are detected, this may have important implications for the future development of core outcome sets [51,52].

#### Study method

##### Delphi study round one

Email addresses for potential participants will be collected by the research team via contacting prostate cancer specific patient-led support groups and charities throughout the United Kingdom (for the patient group), and national and international professional bodies such as BAUS and the European Association of Urologists (EAU) (for the healthcare professionals group). Each participant will be sent an online questionnaire. Their name and email address will be used to generate unique identifiers which enable identification of all participants completing each round of the Delphi study. Participants will be asked to complete the Delphi questionnaire within three weeks and will be prompted after week two with an email reminder. The Delphi questionnaires will contain lay terminology which will be listed alongside clinical terms to assist patients in understanding complex terminology (such as positive surgical margins).

Participants will be required to identify which stakeholder group they belong to using a dropdown menu. In addition, within each stakeholder group there will be further categories to choose from these dropdown menus. For instance, the patient group will be required to identify their age group (older than 60 or younger than 60), the intervention they received (open surgery, EBRT, active surveillance, and so forth), and the time since their intervention (more than one year ago or less than one year ago). Healthcare professionals will be asked to identify their role (specialist nurse, laparoscopic surgeon, robotic surgeon, open radical prostatectomy surgeon, cryotherapist or HIFU specialist, radiation oncologist, brachytherapy oncologist, and so forth).

In round one, the participants will be asked to consider treatment decisions and the benefits and adverse events associated with treatment. Separate instructions and guidance will be provided for healthcare professionals and patients via an online link to allow tailoring of the language (technical versus lay) to describe terms. For the questionnaire, identical questions will be used for healthcare professionals and patients alike: ‘How important are the following outcomes in making decisions regarding prostate cancer treatment?’.

Participants will be asked to score the importance of each of the outcomes listed on a nine-point scale (scores grouped into one to three = not important; four to six = important but not critical; or seven to nine = critical; as well as an ‘unable to score’ option). This scale was devised by the Grading of Recommendations Assessment, Development and Evaluation (GRADE**)** to score the quality of evidence for outcomes in systematic reviews and has been adopted in other core outcome development research groups using Delphi methods [49]. Round one will also provide an option for any participant to add any additional outcomes. Non-responders will not be invited to participate in round two.

##### Analysis of Delphi study round one

Descriptive statistics will summarise the results of round one, including the percentage of participants scoring the outcome at each possible response from one to nine. Any additional outcomes identified by participants that are deemed to represent a new outcome by the researchers (TL and SM) will be included for round two, and any uncertainties will be discussed with the study advisory group. All outcomes will be carried forward to round two.

#### Delphi study round two

Participants will complete round two online. All participants will be reminded of their own round one scores. Participants will be randomised to one of three groups as follows:

1. Group one (consisting of a third of the patient stakeholder group and a third of the healthcare professional stakeholder group, randomly allocated) will be shown the proportion of people in their own stakeholder group choosing each score from one to nine.
2. Group two (consisting of another third of the participants from each stakeholder group, randomly allocated) will be shown the proportion of people in their stakeholder group choosing each score from one to nine and the other stakeholder group’s scores.
3. Group three (consisting of the remaining third from each stakeholder group) will shown both stakeholder groups’ scores combined.

Participants will be asked to re-score the outcomes. All outcomes from round one will be carried forward.

##### Analysis of Delphi study round two

The proportion of participants scoring the outcome at each possible response from one to nine will be used to summarise round two. All outcomes will be carried forward to round three.

##### Delphi study round three

Round three will also be completed online. All participants will be reminded of their round two scores. If response rates number at least 10 per stakeholder group per arm, participants will be retained in their randomised feedback groups one, two, and three, and will only have access to the feedback scores from others randomised to their group. If response rates are poor then the randomised groups one, two, and three will be shown feedback from all other participants, regardless of which group those participants were randomised to. This will be done in order to maximise information. Participants will be asked to re-score the outcomes.

##### Analysis of Delphi study round three

For each outcome presented in round three, the proportion of participants from each stakeholder group scoring one to three, four to six, and seven to nine on the nine-point Likert scale will be calculated for each item regardless of randomised group. ‘Consensus in’ (consensus that the outcome should be included in core set) will be defined as greater than 70% of participants scoring between seven and nine and less than 15% of participants scoring between one and three. ‘Consensus out’ (consensus that the outcome should not be included in core set) will be defined as greater than 70% of participants scoring between one to three and less than 15% of participants scoring between seven and nine. All other combinations will be considered ‘equivocal’. The outcomes that are designated as ‘consensus in’ by both stakeholder groups will be included in the final core outcome set to be carried forward to the consensus meeting. Results for all outcomes will be presented at the consensus meeting, including those designated ‘consensus out’ by both stakeholder groups, in order to remind the stakeholders what the result of the Delphi process was.

The nested RCT will be analysed to ascertain whether participants having seen feedback from their group only, the other group’s feedback only, or their own group’s and the other group’s feedback alters scoring.

#### Consensus group meeting

Research question: Can we derive a core outcome set from the two sets of key outcomes from the stakeholder groups?

##### Study overview

A consensus meeting with key stakeholders (patients and healthcare professionals) will be conducted at the end of the Delphi process. The participants for the consensus group meeting will be purposively sampled to ensure a range of views of men who have had the various treatments and the health professionals that administer them are represented. The sample for both stakeholder groups will be drawn from those who completed all rounds of the Delphi study. All outcomes defined as ‘consensus in’ by both stakeholders will be accepted, all those defined ‘consensus out’ by both stakeholders will be rejected, and all others will be discussed at the consensus meeting. The objective of the consensus meeting is to discuss outcomes about which there was disagreement in round three of the Delphi study, and to validate and agree on a final list of outcomes which will constitute the core outcome set. A secondary objective is to explore how treatment type may affect outcomes regarded as important by patients.

### Ethical arrangements

Ethical approval has been sought and obtained for the project by the National Research Ethics Service (NRES) - North of Scotland Committee (reference 12/NS0042).

## Discussion

There is no published core outcome set for localised prostate cancer effectiveness trials which has been developed in conjunction with key stakeholders using robust, standardised, and transparent methodology. An outcome set for routine hospital data collection, focussed on comparability across institutions and individual clinicians, with a view toward lowering costs has been developed by ICHOM [38,39]. The present research will standardise core outcome definition, collection, measurement, analysis, and interpretation in effectiveness trials for localised prostate cancer. It will propose a core outcome set recommending what outcomes should be measured or at least considered in comparative clinical effectiveness trials. However, importantly, trialists may supplement the core outcomes set with other outcomes, because the set represents a minimum. In addition, there may be situations where the core outcome set may not be relevant, especially for studies with certain specific or focused objectives (for example, a trial designed to ascertain hernia rates via different surgical interventions, or different vesico-urethral anastomotic suturing techniques on duration of drainage). Nevertheless, even under such circumstances, there is an argument for such researchers to consider if the interventions being assessed are likely to have any impact on core outcomes, and if so, then to consider measuring the relevant ones.

This research will benefit all stakeholders involved in prostate cancer management in Scotland, the United Kingdom, and beyond. Specifically, the study will have the following long-term benefits:

1. Choice of treatments can be more fully informed by patients’ needs, and patients will have access to improved services including enhanced decision aids, better counselling, full disclosure of information during informed consent, and shared decision-making.
2. Clinicians will be better equipped to provide informed consent and facilitate decision-making by patients, and be able to foster improvements in clinical governance and in the design of decision aids.
3. The NHS will be better equipped to prioritise funding of localised prostate cancer treatments that reflects the needs of patients.
4. Researchers will be able to design trials and synthesise evidence which address the most important outcomes to all stakeholders, hence encouraging participation of clinicians and patients in clinical trials.

In addition, the output will serve as a platform to develop patient-reported core outcome measures for use in clinical trials of interventions for localised prostate cancer and in clinical practice. The methodology developed will also serve as a model for the development of core outcome measures in other urological conditions and across other surgical specialities. The output will also facilitate further studies designed to better understand patients’ decision-making processes, through the exploration of the weights patients give to alternative outcomes and the trade-offs made when making a treatment choice. The inclusion of key stakeholder groups in all processes of the research ensures that the core outcome set will be relevant to these groups and accepted as useful in future research. The long-term objective is to develop and validate a universal set of core outcome measures relevant to all interventions for all urological cancers.

## Trial status

Participant recruitment for the Delphi study started in December 2014.

## Additional file

Additional file 1: Localised prostate cancer care pathway.

## Abbreviations

3D-CRT: Three-Dimensional Conformal Radiotherapy
BAUS: British Association of Urological Surgeons
COMET: Core Outcome Measures in Effectiveness Trials
EAU: European Association of Urology
EBRT: External Beam Radiotherapy
GRADE: Grading of Recommendations Assessment, Development and Evaluation
HIFU: High Intensity Focussed Ultrasound
ICHOM: International Consortium for Health Outcomes Measurement
IMRT: Intensity Modulated Radiotherapy
NHS: National Health Service
NIHR HTA: National Institute for Health Research Health Technology Assessment
OMERACT: Outcome Measures in Rheumatology
PCSF: Prostate Cancer Support Federation
PSA: Prostate Specific Antigen
RCT: Randomised Controlled Trial
UCAN: Urological Cancer Charity.
