# Supplementary figures and images for: A core outcome set for localised prostate cancer effectiveness trials: protocol for a systematic review of the literature and stakeholder involvement through interviews and a Delphi survey
Source: Trials. 2015 Mar 4;16:76. doi: 10.1186/s13063-015-0598-0 (PMC4355995; doi:10.1186/s13063-015-0598-0)

# Appendix 1. Localised prostate cancer care pathway

## Localised Prostate Cancer

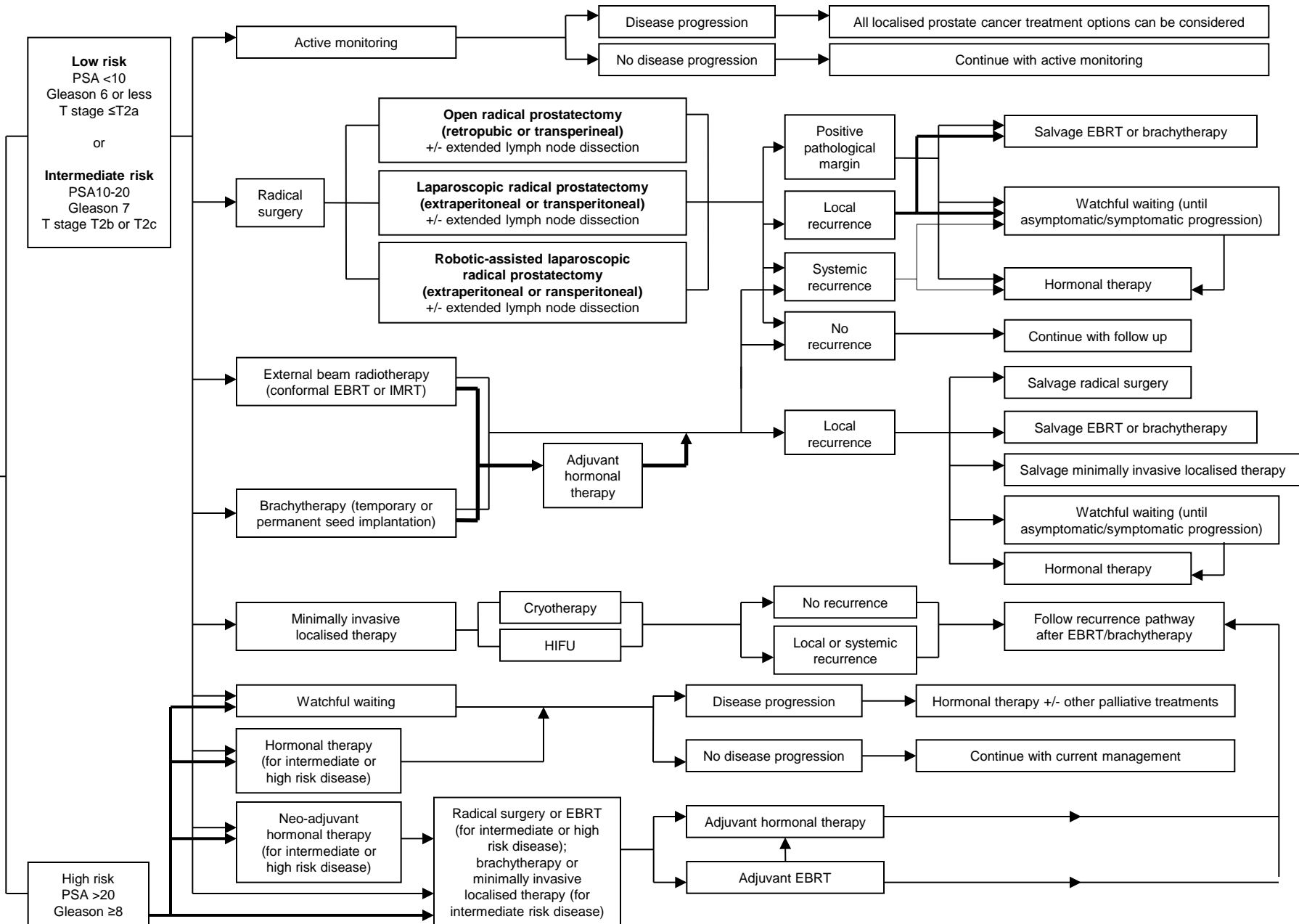

Supplement: Additional file 1: — Localised prostate cancer care pathway. [file 13063_2015_598_MOESM1_ESM.pdf]
